# The reliability of remote photoplethysmography under low illumination and elevated heart rates

**DOI:** 10.1038/s41746-025-02192-y

**Published:** 2025-12-03

**Authors:** Bhargav Acharya, William Saakyan, Barbara Hammer, Hanna Drimalla

**Affiliations:** 1 Human-Centered Artificial Intelligence Group, Bielefeld University, Bielefeld, Germany; 2 Machine Learning Group, Bielefeld University, Bielefeld, Germany

**Keywords:** Cardiology, Computational biology and bioinformatics, Health care, Mathematics and computing

## Abstract

Remote photoplethysmography (rPPG) offers a non-invasive means of estimating heart rate in telemedicine settings. Yet its reliability remains uncertain due to the limited diversity and ecological validity of existing benchmark datasets. In this work, we systematically investigate the robustness of rPPG methods under challenging conditions, specifically low illumination and elevated heart rates. We introduce the CHILL dataset, which comprises video and PPG signals collected from 45 participants across two lighting conditions (bright and dark) and exercise-induced heart rates ranging from 54 to 141 beats per minute. We assessed eight rPPG algorithms, including four signal processing-based and four deep learning-based approaches, across three datasets: the newly collected CHILL dataset and two widely used public benchmarks, PURE and COHFACE. Within-dataset analysis on the CHILL dataset revealed that many existing rPPG methods struggle under challenging conditions. Five of the eight methods experienced a statistically significant decline in performance at elevated heart rates. In contrast, low illumination had a comparatively smaller impact. Cross-dataset analysis further revealed that several deep learning models failed to generalize effectively to the CHILL dataset. Among the models that did generalize, many still showed a significant decline in performance under elevated heart rate conditions, regardless of the training dataset. These findings highlight a critical limitation in current rPPG algorithms, namely their susceptibility to high heart rates. Our evaluation of rPPG methods on the CHILL dataset underscores the need for more robust approaches to enable accurate, non-invasive physiological monitoring in real-world digital health environments.

## Introduction

Non-contact and remote health monitoring solutions promise to enhance patient convenience, alleviate burdens on traditional healthcare infrastructure, and enable vital sign monitoring in diverse, everyday settings, from homes to fitness environments^1–3^. Of the different physiological signals, Heart rate is a fundamental physiological signal and a critical digital biomarker to assess cardiovascular health, autonomic function, stress response, and overall well-being^4^.

Traditionally, heart rate has been monitored using contact methods such as Electrocardiogram (ECG) and Photoplethysmography (PPG). However, Remote Photoplethysmography (rPPG) has emerged as a promising non-contact alternative, particularly relevant for the advancement of digital medicine due to its potential for unobtrusive heart rate estimation. This technique leverages standard cameras to extract physiological signals from subtle changes in skin color caused by changes in blood volume^5^. Over the past decade, significant research efforts have focused on developing various rPPG methods, ranging from signal processing techniques analyzing color channels^5,6^ to more recent approaches leveraging deep learning architectures^7–10^ to implicitly learn the relationship between video input and physiological signals. The advantages of rPPG, including its non-invasiveness, ease of integration into ubiquitous devices such as smartphones and webcams, and potential for monitoring, position it as a key technology for the future of digital medicine.

Despite these advancements and significant promise, the reliable deployment of rPPG in real-world digital medicine applications faces considerable challenges^11,12^. Existing rPPG methods are known to be susceptible to various environmental and physiological factors, including illumination variance^6^, motion artifacts^7^, video compression^9^, and elevated heart rates. Among these, two critical yet underexplored factors are low illumination and elevated heart rates, both of which are highly relevant in digital medicine scenarios.

Most rPPG methods are based on the skin reflectance model^5^, which assumes sufficient ambient lighting to capture pulsating color changes. However, in low-illumination conditions, signal fidelity decreases, and it can be challenging to accurately estimate the rPPG signal. However, widely used rPPG datasets^11,13–18^, are recorded in well-lit environments. As a result, current deep learning models may overfit to ideal conditions and fail to generalize to more challenging real-world use cases.

Similarly, existing rPPG datasets are commonly collected from subjects with resting heart rates, resulting in a narrow distribution of heart rates across recordings^11,13,15–17^. However, elevated heart rates are common among patients in telemedicine. These elevations can result from physical activity, emotional stress, or acute cardiovascular events that include clinically significant conditions such as tachycardia (>100 bpm) and bradycardia (<60 bpm), which are prevalent in both acute and chronic cardiovascular disorders. This narrow distribution of heart rates in rPPG datasets has led to a systematic under-representation of elevated heart rates, which limits the clinical relevance of current benchmarks. This under-representation may further lead to over-fitted models that boast superior results on public datasets that would fail to generalize in real-world settings. A recent review^10^ emphasized this gap, noting that rPPG methods have not been evaluated across various heart rate ranges. It also reported that performance in open challenges such as RePSS 2020^19^ peaked within a narrow heart rate band (77–90 bpm), further underscoring the need for datasets that capture wider heart rate distributions. Some relevant works, such as LGI-PPG^18^ and ECG-Fitness^20^, have introduced elevated heart rates into the dataset, but these were collected during exercise, introducing additional confounding factors that prevent researchers from independently studying the effect of elevated heart rate on the rPPG method performance.

These gaps in systematic evaluation, particularly under low illumination and elevated heart rates, present significant obstacles to the practical deployment of rPPG systems in real-world digital health settings. To advance the field, it is essential to develop datasets and evaluation protocols that more accurately reflect these clinically relevant and often underrepresented conditions.

In this work, we address these limitations through the following key contributions:

1. We introduce the Challenging Heart Rate and Illumination (CHILL) dataset, a novel, carefully curated benchmark comprising synchronized video and PPG signals from 45 participants, captured under two lighting conditions (bright and dark) and two heart rate states (resting and elevated).
2. We conduct a comprehensive evaluation of eight rPPG methods, including four state-of-the-art deep learning-based models and four signal processing-based approaches, to assess their robustness under these challenging conditions.
3. We further evaluate the generalizability of four deep learning-based rPPG methods, trained on public datasets, to low-light conditions and elevated heart rates present in the CHILL dataset.

Together, these contributions lay the groundwork for developing more robust rPPG methods suitable for deployment in real-world digital health and telemedicine settings. rPPG methods can be broadly classified into two main categories: signal processing-based methods and deep learning-based methods. We first provide an overview of rPPG methodologies before presenting our experimental results

Early rPPG methods relied primarily on signal processing-based methods to extract physiological information from video. Verkruysse et al. first demonstrated that the green channel of RGB video could be used to extract rPPG signals for heart rate estimation. This approach leverages the fact that hemoglobin exhibits greater absorption of green light wavelengths compared to red and blue wavelengths^21^.

Subsequent works incorporated the knowledge of how light interacts with the skin into the methods. This interaction of light was first modeled by Wang et al.^5^, who introduced the skin reflection model. This model considers that the light captured by the camera, which is reflected from the skin, consists mainly of two components: specular and diffuse. Specular reflection is the surface-level skin reflection and does not contain relevant HR information. In contrast, diffuse reflection corresponds to light that is reflected from the skin tissue and blood vessels, which contain information on the changing blood volume.

Methods such as CHROM^6^ and POS^5^ were developed to eliminate these extraneous specular reflections. CHROM^6^ does this by considering the differences in the color channels, while POS^5^ uses a projection of the reflected light onto a plane orthogonal to the skin. Other works used statistical dimensionality reduction techniques such as PCA^22^ and ICA^23^ to estimate the rPPG signal.

These methods are considered baseline approaches in the rPPG field, and newly proposed methods are typically evaluated against these established signal processing-based techniques

The emergence of deep learning brought a paradigm shift to rPPG research. Špetlík et al.^20^ were one of the first to show that deep learning-based approaches can be used for the task of rPPG estimation, utilizing 2D-CNNs within their architecture. In addition, they made use of a second network called the extractor to estimate the HR from the predicted rPPG signal. Yu et al.^8^ experimented with methods that incorporated temporal dimensions of the video input. This led to the development of Physnet^8^, which used 3D-CNNs instead of 2D-CNNs.

The success of deep learning-based methods led to the development of specialized architectures addressing specific challenges in rPPG estimation, including motion artifacts and video compression artifacts. DeepPhys, developed by Cheng et al.^7^, specifically targets motion artifacts. The DeepPhys architecture employs a dual-branch design comprising separate motion and appearance pathways, each utilizing 2D-CNNs based on the VGG architecture^24^. An attention mechanism connects these independent branches, directing the model to focus on image regions most relevant to rPPG signal extraction. Liu et al.^25^, similar to DeepPhys^7^, proposed a two-branch architecture that could estimate the respiration rate along with the heart rate. They utilized temporal shift convolutions^26^, which helped reduce the number of training parameters without sacrificing temporal These smaller models can be deployed on mobile platforms with limited processing power, further expanding the accessibility and ubiquity of rPPG technology.information.

Video compression, while essential for data handling, is known to degrade the Videos are generally compressed to make handling them easier; however, compression artifacts and information loss create significant challenges for rPPG extraction algorithms. In this regard, Yu et al. proposed STVEN^9^ and rPPGNet^9^ to mitigate the loss in performance of rPPG methods on highly compressed videos. The STVEN architecture enhances the highly compressed videos, which are then processed by rPPGNet to estimate the rPPG signal. The rPPGNet^9^ utilizes a spatiotemporal convolutional network, which takes in 64 consecutive frames of the input video and outputs the corresponding rPPG signal. Additionally, the model incorporates a skin detection-based attention module to eliminate the influences of non-skin regions of the video.

## Results

This section outlines the experimental outcomes of our study. We begin by presenting CHILL, our novel open-source dataset for rPPG. We then present the results of our systematic evaluations for eight rPPG methods evaluated on existing public datasets and CHILL. This contains two main evaluations. First, we performed within-dataset cross-validation using participant-wise splits to evaluate how well the methods perform when trained and tested on the same dataset. In this scenario, we aimed to assess whether deep learning-based rPPG methods are robust to variations in illumination and the presence of elevated heart rates when such conditions are already represented during training. We similarly evaluated the signal processing-based rPPG methods to investigate their inherent robustness under these conditions. These experiments are detailed in the “Within-dataset performance of signal processing-based and deep learning-based methods” section.

Second, we examine the generalization ability of the deep learning methods by training them exclusively on public datasets and testing them on our collected CHILL dataset. This evaluation explores how well deep learning-based rPPG methods trained on public datasets generalize to changes in illumination and elevated heart rates. Further details are provided in the “Generalization of deep learning rPPG methods” section.

Following each evaluation, we provide a statistical analysis of the influence of illumination (“Effect of illumination on within-dataset performance of rPPG methods” and “Effect of illumination on the generalization performance of rPPG methods” sections) and elevated heart rates (“Effect of elevated heart rate on within-dataset performance of rPPG methods” and “Effect of elevated heart rates on the generalization performance of rPPG methods” sections) on the performance of rPPG methods.

### Challenging heart rate and illumination (CHILL) dataset

To address the limitations of publicly available datasets, which often lack real-world low-illumination conditions and elevated heart rates, we collected a novel dataset called CHILL.

**Fig. 1:**
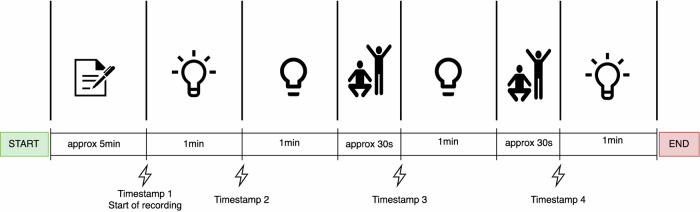
Overview of the CHILL recording protocol. Each session begins with informed consent and equipment setup, followed by a sequence of four one-minute recordings under different lighting and heart rate conditions. Light settings alternate between bright and dim, and heart rate is elevated mid-session using brief physical activity. Time-aligned markers were used for synchronization between video and physiological signals.

**Fig. 2:**
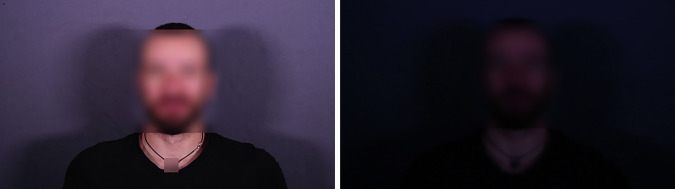
Representative video frames from the CHILL dataset under two illumination conditions. Left: Participant recorded under the bright setting. Right: The same participant under the dark setting.

The CHILL dataset comprises video recordings and corresponding photoplethysmography (PPG) signals from 45 participants (28 females, 17 males), aged 18–32 years (*M* = 24.6). Participants represented Fitzpatrick skin types I, II, and III (on a scale from I to VI). A total of 180 one-minute video recordings were collected under two lighting conditions (bright and dark) and two heart rate states (resting and elevated). Elevated heart rates were induced through physical exercises, including pushups and sit-ups. This systematic variation in lighting and heart rate was carried out to have four distinct settings: Resting-Bright, Resting-Dark, Elevated-Bright, and Elevated-Dark. The data collection protocol is illustrated in Fig. 1, which gives an overview of the data collection procedure. The Video was recorded at a resolution of 1920 × 1080 pixels using a Canon EOS 550D DSLR camera at 25 frames per second (fps). PPG signals were acquired using a finger pulse oximeter, which is a part of the Biosignalplux Explorer Kit, sampled at 1000 Hz. The samples from the bright and dark settings of the collected dataset are shown in Fig. 2.

**Fig. 3:**
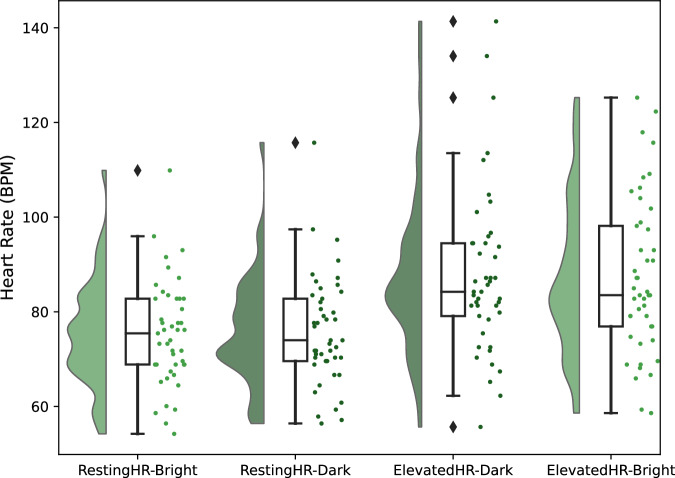
Heart rate distributions across four experimental scenarios within the CHILL dataset. Each violin plot illustrates the distribution, median, and variability of heart rate (in BPM) within each dataset.

Figure 3 illustrates the distribution of heart rates across the four experimental settings. Participants’ heart rates ranged from 54 to 141 beats per minute (bpm). Overall, averaged heart rates were 76.2 bpm for the resting heart rate settings and 87.3 bpm in elevated heart rate settings. To verify that our experimental protocol successfully induced elevated heart rates, we compared the mean heart rates under resting and elevated conditions across all participants. A Wilcoxon signed-rank test revealed a statistically significant difference between the two conditions (*W* = 7.0, *p* < 0.001), with a large effect size (*r* = −0.86) and a median difference of Δ = −9.521 bpm.

Table 1 summarizes key properties of the CHILL and the public rPPG datasets COHFACE^17^, and PURE^13^, including participant demographics, capture settings. CHILL includes more participants than either the public dataset or a higher proportion of female participants.

**Table 1:** Summary of rPPG datasets used in this study, highlighting differences in demographics, recording conditions, and heart rate

Dataset	Participants	Lighting conditions	FPS	Resolution	HR range
COHFACE^17^	40 (F:12, M:28)	Natural and studio	20	640 × 480	45–97
PURE^13^	10 (F:2, M:8)	Natural	30	640 × 480	42–138
CHILL	45 (F:28, M:17)	Studio (bright and dark)	25	1920 × 1080	54–141

**Fig. 4:**
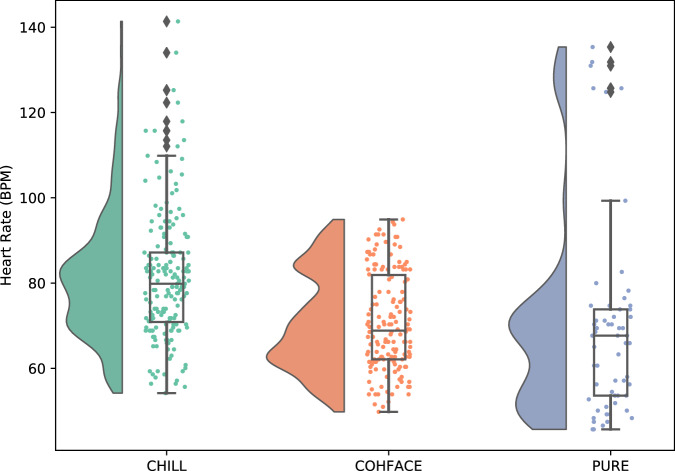
Comparison of heart rate distributions across CHILL, COHFACE^17^, and PURE^13^. Each violin plot illustrates the distribution, median, and variability of heart rate (in BPM) within each dataset.

Figure 4 highlights the difference in heart rate distribution between the collected CHILL dataset and public rPPG datasets. To statistically assess these differences in ground truth heart rate distributions, we performed two-sided Mann–Whitney U tests between CHILL and each public dataset. CHILL exhibited significantly higher heart rates compared to COHFACE (*U* = 20038.5, *p* < 0.001, *rank-biserial correlation* = 0.41) and PURE (*U* = 8062.5, *p* < 0.001, *rank-biserial correlation* = 0.52), indicating a moderate to large effect. Median heart rates were 79.83 bpm for CHILL, compared to 68.85 bpm for COHFACE^17^ and 67.68 bpm for PURE^13^.

**Fig. 5:**
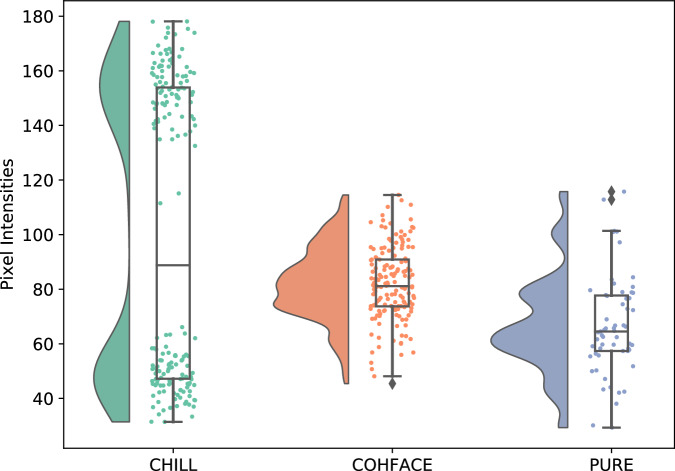
Distribution of mean pixel intensities from face-cropped frames across CHILL, COHFACE^17^, PURE^13^. Each violin plot illustrates the distribution, median, and variability of pixel values within each dataset.

To assess visual brightness and variability across datasets, we computed the mean pixel intensity of each video by first detecting and cropping the face region using the Viola-Jones algorithm^27^, and then averaging pixel values across all frames. Figure 5 shows the distribution of these mean intensities across CHILL, COHFACE^17^, and PURE^13^. The CHILL dataset exhibits a bimodal intensity distribution, reflecting its inclusion of both bright and dark lighting conditions. In contrast, COHFACE and PURE show narrower distributions concentrated in mid-to-low intensity ranges.

### Within-dataset performance of signal processing-based and deep learning-based methods

We conducted our systematic evaluation on two public datasets (COHFACE^17^ and PURE^13^) and our collected dataset, CHILL. The four deep learning-based methods were evaluated using a 10-fold cross-validation strategy with a participant-wise split. The four signal processing-based methods were directly evaluated on the datasets. Mean absolute error (MAE) is reported for all methods, and the results of these evaluations are summarized in Table 2.

**Table 2:** Performance (MAE ± SE, in bpm) of methods across all datasets

Method	PURE	COHFACE	CHILL
DeepPhys^7^	**3.1** ± **2.1**	4.4 ± 1.3	9.1 ± 2.3
TS-CAN^25^	10.1 ± 5.5	4.1 ± 2.1	3.2 ± 0.2
PhysNet^8^	4.0 ± 1.9	**1.6** ± **0.4**	4.1 ± 1.3
rPPGNet^9^	8.8 ± 4.5	3.4 ± 1.8	2.0 ± 0.4
GREEN^21^	10.1 ± 2.9	7.1 ± 0.7	2.6 ± 0.6
CHROM^6^	8.9 ± 2.1	10.2 ± 0.6	1.8 ± 0.6
POS^5^	7.5 ± 2.0	11.8 ± 0.7	**1.1** ± **0.1**
ICA^23^	4.9 ± 2.0	7.4 ± 0.6	5.4 ± 0.9
Dummy	15.6 ± 2.2	9.7 ± 0.7	10.8 ± 0.7

Deep learning-based methods (top) are evaluated using 10-fold cross-validation with participant-wise splits. Signal processing-based methods (bottom) are evaluated on the whole datasets. A dummy estimator (bottom) that always predicts the mean heart rate of the training set is included as a baseline. Bold values indicate the lowest MAE (best performance) for each dataset.

*SE* standard error.

The performance of the eight rPPG methods varied across the datasets. Deep learning-based rPPG methods outperformed signal processing-based methods on all existing public datasets (PURE^13^ and COHFACE^17^). For instance, DeepPhys^7^ and PhysNet^8^ achieved the lowest MAE on PURE^13^ and COHFACE^17^, respectively. However, on the CHILL dataset, signal processing-based method POS^5^ outperformed all other methods, with CHROM^6^ demonstrating competitive performance.

### Effect of illumination on within-dataset performance of rPPG methods

To investigate the influence of illumination on the performance of rPPG methods, we systematically compare the changes in performance of rPPG methods under different illumination conditions present in CHILL and COHFACE^17^.

**Fig. 6:**
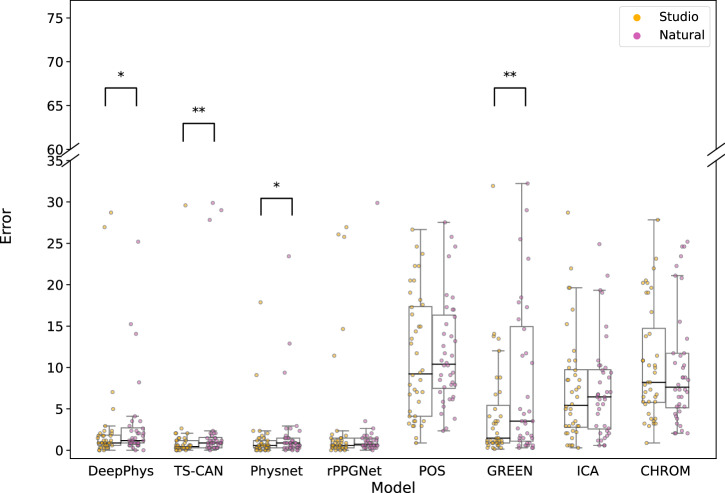
Distribution of Error (MAE) under studio and natural lighting of the COHFACE dataset for all rPPG methods. Each point overlaid on the box plots represents the MAE for an individual participant under the corresponding lighting condition. Statistical significance between the two lighting conditions was assessed using the Wilcoxon signed-rank test. See Supplementary Table 1 for the corresponding test statistics.

For COHFACE^17^, we performed a Wilcoxon signed-rank test comparing the MAEs under studio and natural lighting for all methods. The quantitative results of these paired comparisons are presented in Supplementary Table 1, and the visualization is provided in Fig. 6. Among the deep learning-based methods, DeepPhys^7^(*p* = 0.033), Physnet^8^ (*p* = 0.0307), and TS-CAN^25^ (*p* = 0.0062) showed statistically significant differences, all three exhibiting better performance (negative Δ median difference) under studio conditions. rPPGNet^9^, however, did not show a statistical difference *p* = 0.3642, in performance. GREEN^21^ was the only signal processing-based method that showed a statistically significant difference (*p* < 0.001) with a negative median difference (Δ Median = −0.879), indicating better performance under studio lighting conditions. In contrast, other signal processing-based methods, ICA^23^, POS^5^, and CHROM^6^ did not show statistically significant differences between the two illumination settings (*p* > 0.05 for all comparisons), implying that their performance on COHFACE^17^ was not significantly affected by changes in illumination.

**Fig. 7:**
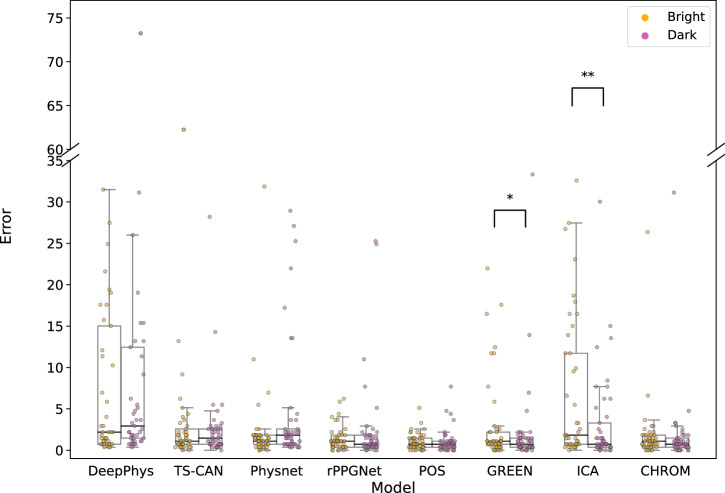
Distribution of Error (MAE) under bright and dark lighting of the CHILL dataset for all rPPG methods. Each point overlaid on the box plots represents the MAE for an individual participant under the corresponding lighting condition. Statistical significance between the two lighting conditions was assessed using the Wilcoxon signed-rank test. See Supplementary Table 2 for the corresponding test statistics.

A similar analysis was performed on the CHILL dataset, comparing model performance under bright and dark lighting conditions. The corresponding results are summarized in Supplementary Table 2 and visually represented in Fig. 7. Only signal processing-based methods, GREEN^21^ (*p* = 0.045) and ICA^23^ (*p* = 0.0028), showed statistically significant differences. The positive median difference (Δ Median = 0.3662 for GREEN^21^ and Δ Median = 0.732 for ICA^23^) suggests that the model performed better under darker lighting conditions.

### Effect of elevated heart rate on within-dataset performance of rPPG methods

**Fig. 8:**
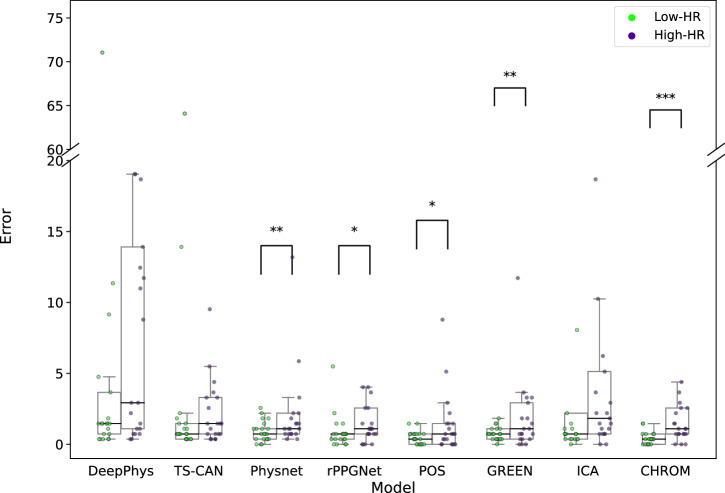
Distribution of errors for Low-HR (<80) and High-HR (>80) settings of the CHILL dataset for all rPPG methods. Each point overlaid on the box plots represents the MAE for an individual participant under the corresponding heart rate condition. Statistical significance between the two heart rate conditions was evaluated using the Wilcoxon signed-rank test. See Supplementary Table 3 for the corresponding test statistics.

We next investigated the influence of participant heart rate on the performance of the rPPG methods. This evaluation was performed on the collected CHILL dataset. To carry out systematic investigations, we filtered participants based on their ground truth heart rate. Specifically, we selected participants (*n* = 21) who had an average heart rate of less than 80 bpm (Low-HR) in the resting scenario and an average heart rate greater than 80 bpm (High-HR) in the elevated scenario of the CHILL dataset. We then analyzed the difference in performance using a one-sided Wilcoxon signed-rank test on this filtered set of participants. Both quantitative and qualitative results are reported in Supplementary Table 3 and Fig. 8, respectively.

Among deep learning methods, Physnet^8^ (*p* = 0.008) and rPPGNet^9^ (*p* = 0.0385) demonstrated significantly better performance in the Low-HR scenario (Δ Medians − 0.733 and −0.366 for Physnet^8^ and rPPGNet^9^, respectively). Similarly, signal processing-based methods GREEN^21^ (*p* = 0.009; Δ = −0.366), POS^5^ (*p* = 0.0427; Δ = 0.0), and CHROM^6^ (*p* < 0.001; Δ = −0.732) showed significantly better performance in the Low-HR scenarios.

### Generalization of deep learning rPPG methods

To assess the generalizability of deep learning models trained on publicly available datasets to challenging scenarios present in our dataset, we train deep learning methods on public datasets while reserving the complete CHILL dataset for testing. The per-scenario MAEs are presented in Table 3.

**Table 3:** Cross-dataset generalization performance of deep learning models on the CHILL dataset

Trained On	Models	Resting		Elevated		Overall
		Bright	Dark	Bright	Dark	MAE
COHFACE^17^	DeepPhys^7^	0.58	1.27	4.51	4.98	2.84
	TS-CAN^25^	**0.44**	**0.60**	1.87	**1.38**	**1.07**
	Physnet^8^	6.69	4.17	15.90	4.70	7.86
	rPPGNet^9^	14.47	27.59	9.24	20.96	18.06
PURE^13^	DeepPhys^7^	0.50	0.67	**1.61**	2.72	1.38
	TS-CAN^25^	0.42	0.94	1.80	1.44	1.15
	Physnet^8^	12	9.99	20.63	19.67	15.58
	rPPGNet^9^	8.95	19.06	18.08	14.90	15.25
	Dummy	9.62	9.81	12.41	11.25	10.75

Models were trained on public datasets (COHFACE^17^ and PURE^13^) and evaluated on CHILL. Performance measured as MAE (bpm). Bold values highlight the best performance for each setting.

Overall, TS-CAN^25^ demonstrated the most robust performance on the CHILL dataset, achieving the lowest MAEs across most conditions when pretrained on COHFACE^17^. While DeepPhys^7^ also showed relatively lower MAEs compared to PhysNet^8^ and rPPGNet^9^, its performance was not consistently the best. Notably, models pretrained on COHFACE^17^ generally exhibited better generalization to the CHILL dataset compared to those pretrained on PURE^13^, with the exception of DeepPhys^7^. However, both TS-CAN^25^ and DeepPhys^7^ had better performance in the resting condition compared to the elevated heart rate condition.

### Effect of illumination on the generalization performance of rPPG methods

**Fig. 9:**
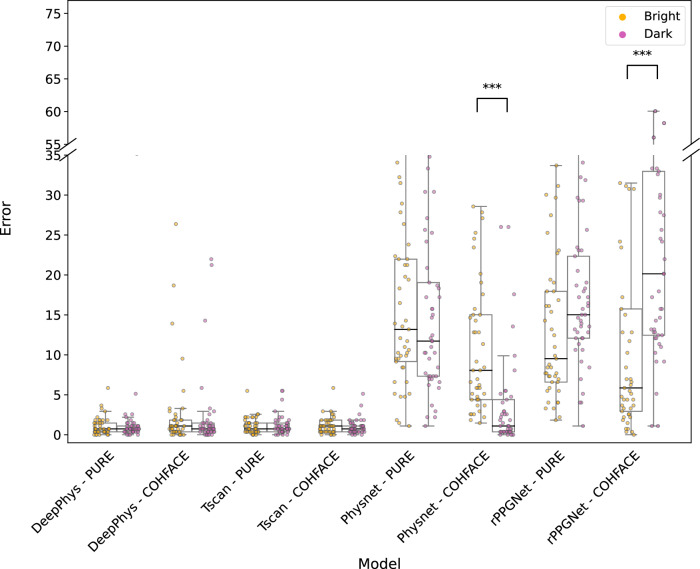
Distribution of Error (MAE) under bright and dark lighting of the CHILL dataset for all pretrained rPPG methods. Each point overlaid on the box plots represents the MAE for an individual participant under the corresponding lighting condition. Statistical significance between the two lighting conditions was assessed using the Wilcoxon signed-rank test. See Supplementary Table 4 for the corresponding test statistics.

Following the same statistical approach as in our analysis of all rPPG methods under varying illumination (“Within-dataset performance of signal processing-based and deep learning-based methods” section), we report the results of the Wilcoxon signed-rank test for all deep learning methods trained on public datasets (PURE^13^ and COHFACE^17^) in Supplementary Table 4. For the methods trained on the PURE^13^, none showed a statistically significant difference in performance between the bright and dark illumination conditions. In contrast, Physnet^8^ (*p* < 0.001) and rPPGNet^9^(*p* < 0.001), pretrained on the COHFACE^17^ exhibited a statistically significant difference in performance under varying illumination, with Physnet^8^ performing better in the Dark scenario and rPPGNet^9^ performing better in the Bright scenario. However, it is important to note that these same methods also demonstrated a large overall MAE when evaluated on the CHILL dataset (as presented in Table 3), indicating poor generalizability as they struggled to accurately predict heart rate on the unseen CHILL data. Figure 9 visually illustrates these differences.

### Effect of elevated heart rates on the generalization performance of rPPG methods

**Fig. 10:**
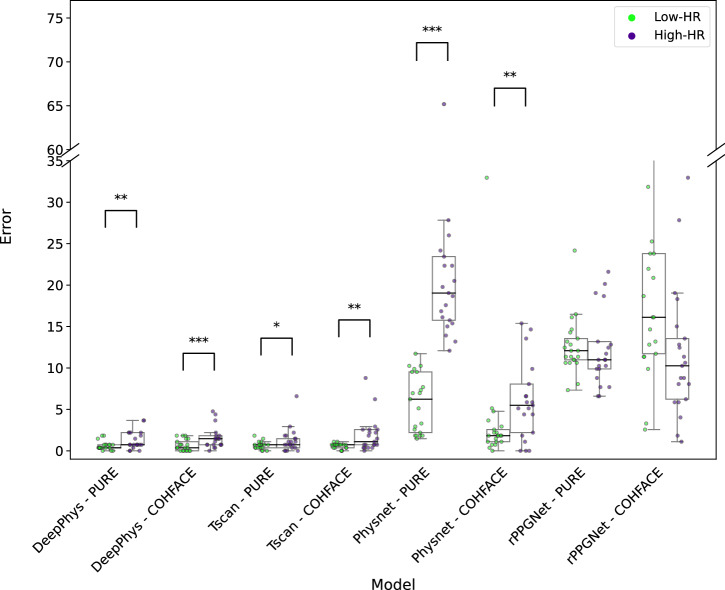
Distribution of errors for Low-HR (<80) and High-HR (>80) settings of the CHILL dataset for all pretrained rPPG methods. Each point overlaid on the box plots represents the MAE for an individual participant under the corresponding heart rate condition. Statistical significance between the two heart rate conditions was evaluated using the Wilcoxon signed-rank test. See Supplementary Table 5 for the corresponding test statistics.

Finally, we investigated the influence of participant heart rate on the performance of the deep learning-based rPPG methods. The results of the one-sided Wilcoxon signed-rank tests for these comparisons are presented quantitatively in Supplementary Table 5 and visualized in Fig. 10.

When trained on PURE^13^, DeepPhys^7^ (*p* = 0.0040), TS-CAN^25^ (*p* = 0.0246), and PhysNet^8^ (*p* < 0.001) exhibited statistically significant performance differences across HR conditions. Methods with statistically significant differences had worse performance in high heart rate conditions. Notably, PhysNet^8^ showed a substantial median shift (Δ Median = −13.55), indicating pronounced sensitivity to high heart rate. In contrast, rPPGNet^9^ did not show a significant difference (*p* = 0.5758).

For models trained on COHFACE^17^, DeepPhys^7^ (*p* < 0.001), TS-CAN^25^ (*p* = 0.0016), and PhysNet^8^ (*p* = 0.0062) again demonstrated a significant decrease in performance in the high heart rate condition. rPPGNet^9^, however, showed no significant difference (*p* = 0.9864).

## Discussions

This study aimed to evaluate the performance and robustness of various rPPG methods, particularly under challenging conditions often underrepresented in existing datasets. To achieve this, we introduced CHILL, a novel dataset incorporating low-illumination scenarios and, crucially, elevated heart rates (Figs. 1 and 3). Our systematic analysis demonstrates that the performance and robustness of rPPG methods are highly context-dependent, particularly in relation to illumination and heart rate variation. We revealed critical limitations in existing rPPG approaches that are often overlooked in traditional benchmarks.

For real-world telemedicine applications, system developers often rely on state-of-the-art implementations that have shown strong performance on publicly available datasets. This approach assumes that high performance on standard benchmarks translates to general reliability. However, our cross-validation results (Table 2) challenge this assumption. We found that no single method consistently performed best across different datasets and conditions. While deep learning methods generally outperformed signal processing-based ones on the public PURE^13^ and COHFACE^17^ datasets, this trend reversed on our CHILL dataset. Here, the signal processing-based method POS^5^ achieved the lowest overall, significantly outperforming all deep learning methods. This underscores the significant influence of dataset characteristics on perceived model performance. These findings show that the effectiveness of an rPPG method is closely tied to the characteristics of the dataset it is applied to. For this reason, developers should not rely solely on results from standard benchmarks. Instead, they should consider the specific scenarios their systems will encounter and evaluate models using representative data before deployment.

Low illumination is common in real-world telemedicine settings and can adversely affect the performance of rPPG methods. Our systematic investigation reveals a more nuanced understanding of its impact. On COHFACE, which contrasts natural and studio lighting, several methods (DeepPhys^7^, TS-CAN^25^, PhysNet^8^, GREEN^21^) showed statistically significant performance degradation under natural lighting compared to studio conditions (Supplementary Table 1, Fig. 6). This aligns with expectations that uncontrolled, fluctuating natural light poses a challenge. However, on the CHILL dataset, where we compared controlled bright and dark studio lighting, most methods demonstrated robustness, with only GREEN^21^ and ICA^23^ showing a statistically significant (though small in median difference) preference for darker conditions (Supplementary Table 2, Fig. 7). This difference between datasets suggests that the nature of illumination variation (controlled vs. uncontrolled) might be more critical than the absolute illumination level within typical indoor settings. While some research has explored challenges like partial face illumination^28^, indicating that deep learning-based models underperform in the presence of low lighting conditions, our results under controlled full-face illumination suggest many methods can handle static low-light conditions reasonably well.

Another relevant yet often underrepresented factor is elevated heart rate, which we investigate in this work. Its impact proved to be a more significant and consistent challenge across rPPG methods. Our analysis on CHILL (Supplementary Table 3, Fig. 8) revealed that performance significantly degraded for the majority of tested methods (PhysNet^8^, rPPGNet^9^, GREEN^21^, POS^5^, CHROM^6^) when participants exhibited heart rates above 80 bpm compared to lower rates. This trend is consistent with previous findings by van der Kooij and Naber^29^, who reported that rPPG methods tend to underperform when heart rates exceed 90 bpm. The agreement across studies suggests a fundamental limitation in how these methods handle elevated heart rates, which are commonly observed in real-world contexts. This finding validates the inclusion of High-HR scenarios in CHILL as a critical factor often missing in standard benchmarks^10^. It highlights a substantial limitation for practical deployment, as many target applications in telehealth for rPPG (e.g., stress detection, monitoring during illness) involve non-resting heart rates.

The challenges associated with elevated heart rate were further amplified in our generalization experiments (Table 3). Critically, the statistical analysis of generalization performance (Supplementary Table 4, Fig. 10) showed that the pretrained models consistently struggled much more in the High-HR conditions within CHILL. This observation stands in contrast to several studies that report strong generalization performance across datasets, often suggesting that the rPPG estimation is largely solved in terms of robustness to domain shifts^7,10,25^. These claims are typically based on cross-dataset evaluations of resting heart rate conditions, where heart rate remains within a narrow range. However, our findings highlight that even models demonstrating overall generalization success may exhibit performance degradation under high heart rate conditions. This underperformance reveals a critical gap in current evaluation practices. Therefore, it is essential that future work incorporates physiologically diverse test conditions, including elevated heart rate scenarios, to ensure that generalization claims are not limited to narrow heart rate ranges.

There are several ways in which the broader applicability of the findings in this work can be improved. The CHILL dataset contains individuals represented by Fitzpatrick skin types I-III, which constrains the generalizability to populations with darker skin tones, which can introduce additional challenges for rPPG-based monitoring. Future iterations of the dataset will aim to incorporate broader skin tone representation to support more inclusive evaluation. Additionally, the dataset contains subjects aged 18–32 years, limiting the characterization to a narrower range of skin textures and physiological properties. Age-related skin changes include significant dermis thinning^30^, which can affect rPPG signal morphology, limiting the generalizability of methods developed primarily on younger populations.

Although CHILL incorporates controlled low-light and elevated heart rate conditions, it does not capture the full range of real-world variability. For future work, it would be beneficial to broaden the scope to include additional artifacts such as motion during physical activity or fluctuations in ambient lighting, which are common in consumer health settings. Furthermore, the current one-minute recording duration limits analysis of recovery dynamics of heart rate and other relevant biomarkers, such as heart rate variability, which has clinical applications, for example, in the diagnosis of depression^31^. Recording longer videos would enable these important analyses.

Our current work uses a Canon 550D DSLR, which has a larger sensor size and better low-light performance compared to those of sensors used in smartphones or webcams. The performance of rPPG methods in different lighting conditions should also be further evaluated with smaller, more ubiquitous sensors.

The analysis of HR effects was restricted to a subset of participants (*n* = 21) with data spanning both low and high HR ranges, limiting the statistical power and robustness of these findings. Finally, given the rapidly evolving nature of deep learning-based rPPG methods^10^ and signal processing-based methods^12,18^, it is important that new approaches continue to be evaluated with this dataset.

Our study highlights the critical influence of low illumination and elevated heart rate on the performance and robustness of rPPG methods. By leveraging the CHILL dataset, which includes carefully designed low-light and elevated heart rate conditions, we uncovered key limitations of current rPPG methods that standard benchmarks often fail to reveal. Notably, no single method consistently excelled across all conditions, underscoring the importance of context-aware evaluation. Elevated heart rates, in particular, pose a significant and consistent challenge for existing approaches, emphasizing the need for broader heart rate ranges in testing. Furthermore, we identified a key limitation in current evaluation practices that generalization claims are frequently based on narrow physiological ranges and may not hold under more diverse real-world conditions. To support reliable deployment in telehealth applications, future research must prioritize validation across physiologically diverse scenarios, including elevated heart rates and other real-world challenges.

## Methods

### Data collection

The data collection procedure, illustrated in Fig. 1, started with an initial 5-min preparatory phase for participant consent and system setup. This was followed by four one-minute recordings per participant under distinct combinations of lighting and heart rate conditions in a controlled laboratory environment. The study was approved by the ethics committee of Bielefeld University. Video and ground truth data collection began at Timestamp 1.

**Fig. 11:**
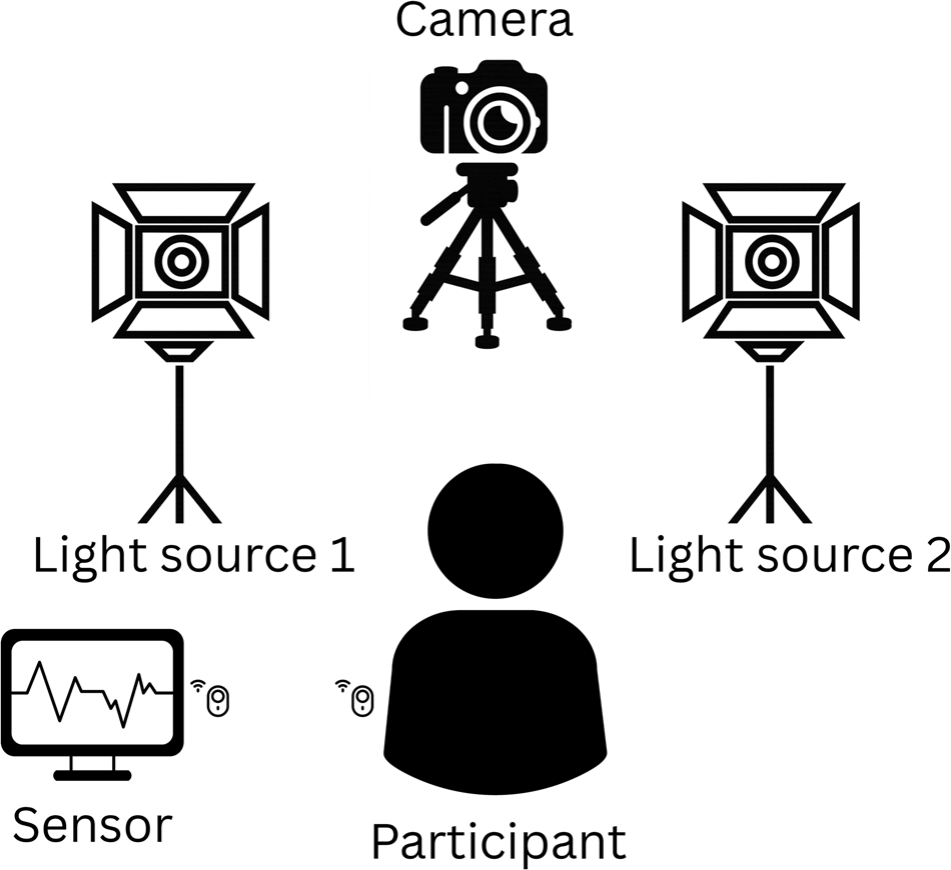
CHILL data collection setup. The participant was seated in front of a high-resolution camera flanked by two adjustable LED light sources. A finger clip sensor collects PPG signals. All signals were recorded and synchronized using a biosignal acquisition system.

The physical arrangement of the sensors and lighting used for data acquisition is shown in Fig. 11. Participants were seated in front of a camera and remained still during the recordings. A clip-on PPG sensor was attached to the left index finger, and ECG electrodes were placed on the chest to collect physiological ground truth data.

To control lighting, two LED array light sources (Dörr 373450 LED Continuous Light DLP-820) were used. Light output was set to 5 (140 lux) for dark conditions and 50 (830 lux) for bright conditions, using a digitally controlled scale of 0–50. Lux measurements were obtained using a VOLTCRAFT VC-8314265 LX-10 Luxmeter. The windows in the room where data collection was carried out were shielded to prevent natural light from influencing the recordings. To induce elevated heart rates, participants performed short exercises (e.g., pushups or squats) before resuming the seated position for recording. This resulted in two scenarios with elevated HR under both lighting conditions. To reduce order effects, the lighting order was randomized for each participant. Synchronization between the video and sensor data was achieved using timestamps recorded at regular intervals by the sensor software. The initial software timestamp was captured using OpenSignals (https://www.pluxbiosignals.com/pages/opensignals) at the start of camera recording. Additional timestamps were recorded prior to the start of each scenario to ensure precise alignment. This enabled post-hoc alignment between video and physiological signals.

The sex of each participant was not collected during the data acquisition phase. Instead, it was inferred post-hoc by two independent researchers who reviewed the recorded videos and annotated the participant’s sex. The annotations from both researchers were fully consistent, showing 100% agreement. This approach was reviewed and approved by the local ethics committee of Bielefeld University.

### Signal processing-based methods

We evaluated four well-established, signal processing-based rPPG algorithms: GREEN^21^, POS^5^, CHROM^6^, and ICA^23^. These methods have been widely adopted as baseline approaches in the rPPG literature due to their simplicity, interpretability, and historical significance in benchmark comparisons. They rely on explicit signal processing pipelines that extract subtle color changes from facial videos using combinations of chrominance-based filtering (CHROM^6^), independent component analysis^23^, or projection methods (POS^5^) to isolate the pulse signal.

All signal processing-based methods were implemented using the rPPG-Toolbox^32^, an open-source Python framework designed for reproducible rPPG research. This framework provides integrated modules for face detection and cropping steps in the rPPG pipeline. In this work, we applied face cropping using the Viola-Jones algorithm^27^ and then downsampled the cropped facial regions to 72 × 72 pixels. For each frame, we calculated the spatial mean of the red, green, and blue color channels within the cropped face region to produce a time series signal, which was then used as the input to each rPPG estimation algorithm. Each method then processed this RGB signal to estimate an rPPG signal.

### Deep learning-based rPPG methods

In addition to signal processing-based approaches, we evaluated four deep learning methods for end-to-end rPPG estimation: PhysNet^8^, DeepPhys^7^, TS-CAN^25^, and rPPGNet^9^. Like the signal processing-based baselines, these deep learning methods are commonly used as reference points in the literature when introducing new state-of-the-art approaches. PhysNet^8^ employs 3D convolutional neural networks to directly extract spatiotemporal features from facial videos for rPPG estimation. DeepPhys^7^ utilizes a two-branch 2D convolutional network that combines spatial and temporal features through an attention mechanism to capture meaningful information relevant for rPPG. Building on DeepPhys, TS-CAN^25^ introduces temporal shift convolutions to more effectively model temporal dynamics across video frames. Finally, rPPGNet^9^ integrates spatiotemporal convolutional networks to learn robust representations. rPPGNet^9^ was introduced in conjunction with STVEN^9^, which enhanced the rPPG signal from highly compressed videos. However, in this work, we focus on the rPPGNet^9^. An overview of these models, including their original training datasets, is reported in Table 4.

**Table 4:** Architectural overview of deep learning-based rPPG methods

Methods	Network	Key innovation	lr
DeepPhys^7^	2D-CNN	Two-branch architecture with an attention mechanism combining spatial appearance and temporal motion features	1.0
TS-CAN^25^	TS-CNN	Builds upon DeepPhys architecture with temporal shift convolutions for improved temporal modeling efficiency	1.0
PhysNet^8^	3D-CNN	End-to-end 3D convolutional network directly extracts spatiotemporal features from facial videos	1e-4
rPPGNet^9^	3D-CNN	Spatiotemporal convolutional network with skin segmentation module for enhanced signal extraction	1e-4

The table summarizes key algorithmic innovations and training configurations used in the original implementations. Face detection via Viola-Jones^27^ was employed by all methods except TS-CAN.

The preprocessing pipelines varied depending on the input requirements of each model. For DeepPhys^7^ and TS-CAN^25^, each frame in the video was downsampled to 36 × 36 pixels for the appearance branch. We maintained this exact preprocessing specification from the original papers to ensure faithful reproduction of the published methods. Subsequent investigation by Nguyen et al.^33^ confirmed that this downsampling does not substantially impact performance for these specific architectures. For the motion branch, temporal normalization was applied using adjacent frames, calculated as:

$$c\,(t)=\frac{c\,(t+1)-c\,(t)}{c\,(t+1)+c\,(t)}$$

where *c*(*t*) denotes the video frame at time *t*. For PhysNet^8^ and rPPGNet^9^, frames were cropped using the Viola-Jones face detector^27^ and resized to 128 × 128 pixels. Furthermore, rPPGNet^9^ was used in masks as an auxiliary input, which were generated using the open-source *Bob* package (https://gitlab.idiap.ch/bob/bob.ip.skincolorfilter) with a threshold of 0.3.

All DL models were trained on NVIDIA A40 GPUs. The *rPPG-Toolbox*^32^ was used to implement TS-CAN^25^, DeepPhys^7^, and PhysNet^8^, while the original codebase from the authors was adapted to integrate rPPGNet^9^ into the evaluation pipeline.

Training protocols were kept consistent across all datasets. Optimizers and learning rate schedules followed those specified in the original implementations, while batch sizes were adjusted according to available GPU memory. No additional hyperparameter tuning was carried out.

Loss functions varied by model: DeepPhys^7^ and TS-CAN^25^ optimized the mean squared error (MSE) between predicted and ground truth signals, while PhysNet^8^ and rPPGNet^9^ minimized the negative Pearson correlation. Notably, rPPGNet^9^ also incorporated binary cross-entropy loss for its skin segmentation module. Readers are referred to the respective original publications for comprehensive details on each loss formulation.

### Heart rate estimation

Following the estimation of the rPPG signal (via signal processing-based or deep learning methods), each signal was filtered using a bandpass filter to suppress noise outside the heart rate frequency range. Subsequently, the heart rate was computed from the power spectral density (PSD) of the filtered signal.

While the *rPPG-Toolbox* defaults to periodogram-based PSD estimation, we employed Welch’s method^34^ instead, as it provides reduced variance in the PSD estimate through segment averaging. The frequency corresponding to the peak power in the PSD was used to derive the estimated heart rate (HR).

Similarly, we estimated the ground truth heart rate for each video using the finger-collected PPG signal. The raw signal was filtered using a bandpass filter. Subsequently, PSD was estimated using the Welch method^34^. The frequency corresponding to the peak power was used as the ground truth heart rate.

### Evaluation metric

We assessed heart rate estimation using the mean absolute error (MAE) metric, defined as:

$$\,{\mbox{MAE}}\,=\frac{1}{S}\mathop{\sum}\limits_{i=1}^{S}\left\vert {{{\rm{HR}}}}_{{{\rm{GT}}}}^{i}-{{{\rm{HR}}}}_{{{\rm{EST}}}}^{i}\right\vert$$

where HR_GT_ and HR_EST_ represent the ground truth and estimated heart rates, respectively, across *S* video samples.

As a baseline, we included a dummy estimator that predicted a constant HR equal to the mean of the training set, providing a simple reference for evaluating the relative performance of the models.

### Statistical analysis

Statistical analyses of heart rate estimation errors were performed using the Wilcoxon signed-rank test (SciPy^35^), a non-parametric method suitable for paired data. The distribution of errors was not normally distributed, as confirmed by the Shapiro–Wilk tests (*p* < 0.05).

#### Impact of low illumination

To assess the influence of illumination, MAEs were calculated per participant for both bright and dark conditions and averaged per scenario. Our null hypothesis (*H*_0_) was that the error distributions would not differ across illumination settings. The alternative hypothesis (*H*_*a*_) was that there would be a statistically significant difference would exist. A two-sided Wilcoxon signed-rank test was used to compare paired MAE values, assessing whether the median difference in MAE between the two lighting conditions was significantly different from zero.

#### Impact of elevated heart rate

We also examined the effect of heart rate on model performance by dividing the data into recordings with heart rates below 80 bpm (Low-HR) and above 80 bpm (High-HR). This threshold was chosen as a midpoint between typical bradycardia (<60 bpm) and tachycardia (>100 bpm). Only participants with data in both ranges (*n* = 21) were included. The MAEs were calculated per participant for both the Low-HR and High-HR conditions and averaged per scenario. The Low-HR group had a mean heart rate of 72.6 ± 5.0 bpm, with a median heart rate of 73.6 bpm (range 58.2–78.4 bpm). The High-HR group had a mean heart rate of 89.4 ± 11.4 bpm, with a median heart rate of 86.4 bpm (range 81.3–133.3 bpm). The null hypothesis (*H*_0_) stated no difference in error distributions between the two heart rate conditions. Based on prior literature suggesting higher errors at elevated heart rates^10,29^, our directional alternative hypothesis (*H*_*a*_) was that the median of the differences in estimation errors would be significantly greater in the >80 bpm group (i.e., MAE_>80*bpm*_ > MAE_<80*bpm*_). A one-sided Wilcoxon signed-rank test was used to test this directional hypothesis.

## Supplementary information

Supplementary information

## Data Availability

The participants were provided with a consent form that asked them for the publication of their dataset. The collected data is made available with anonymization. The anonymization consists of downsampling each frame in the video to 36 × 36 pixels, as used in the experiments. The data of all the participants who have consented to share their data is available online through zenodo.org at 10.5281/zenodo.14637544.

## Code availability

The code supporting the findings of this study is available at: https://github.com/B-Acharya/rPPG-Toolbox/releases/tag/npj-dm.
