# Supplementary material for: The reliability of remote photoplethysmography under low illumination and elevated heart rates
Source: NPJ Digit Med. 2025 Dec 3;8:744. doi: 10.1038/s41746-025-02192-y (PMC12678791; doi:10.1038/s41746-025-02192-y)
Supplement: Supplementary file 1 — Supplementary information [file 41746_2025_2192_MOESM1_ESM.pdf]

Supplementary Table 1 Wilcoxon Signed-Rank Test results for rPPG methods under different illuminations in the COHFACE dataset. For each method, the median error under each condition is reported along with the median of the paired differences (Δ Median), test statistic (W), P-value, and effect size (r).

**Model**	**Median Error**		**Δ Median**	**W-statistic**	**P-value**	**Effect Size (r)**
	**Studio**	**Natural**				
DeepPhys [7]	0.88	1.17	-0.586	197.5	.0330*	-0.4516
TS-CAN [25]	0.44	0.88	-0.293	139.0	.0062**	-0.5759
Physnet [8]	0.59	0.88	-0.293	150.0	.0307*	-0.5526
rPPGNet [9]	0.59	0.73	-0.000	202.0	.3642	-0.4421
POS [5]	9.23	10.40	-1.758	298.5	.2014	-0.2370
GREEN [21]	1.46	3.52	-0.879	178.5	.0090**	-0.4920
ICA [23]	5.42	6.45	-1.172	344.5	.3786.	-0.1392
CHROM [6]	8.20	7.62	0.292	304.5	.3383	-0.2242

$^{*}p < 0.05$

$^{**}p < 0.01$

Supplementary Table 2 Wilcoxon Signed-Rank Test results for rPPG methods under different illuminations in the CHILL dataset. For each method, the median error under each condition is reported along with the median of the paired differences (Δ Median), test statistic (W), P-value, and effect size (r).

**Model**	**Median Error**		**Δ Median**	**W-statistic**	**P-value**	**Effect Size (r)**
	**Bright**	**Dark**				
DeepPhys[7]	2.20	2.93	0.000	466.0	.9326	-0.0867
TS-CA [25]	1.10	1.46	0.000	373.5	.6215	-0.2423
Physnet [8]	1.10	1.83	-0.366	301.0	.0920	-0.3643
rPPGNet [9]	1.10	0.73	0.366	385.0	.7351	-0.2230
POS [5]	0.73	0.73	0.000.	266.0	.4186	-0.4232
GREEN [21]	1.10	0.73	0.366	248.0	.0455*	-0.4535
ICA [23]	1.83	0.73	0.732.	226.0	.0028**	-0.4905
CHROM [6]	1.10	0.73	0.366	361.5	.6882	-0.2625

$^{*}p < 0.05$

$^{**}p < 0.01$

Supplementary Table 3 Wilcoxon Signed-Rank Test results for rPPG methods under different HR ranges in the CHILL dataset. For each method, the median error under each condition is reported along with the median of the paired differences (Δ Median), test statistic (W), P-value, and effect size (r).

**Model**	**Median Error**		**Δ Median**	**W-statistic**	**P-value**	**Effect Size (r)**
	**Low-HR**	**High-HR**				
DeepPhys [7]	1.46	2.93	-1.099	71.0	.0607	-0.3375
TS-CAN [25].	0.73	1.46	-0.366	53.0	.2184	-0.4740
Physnet [8]	0.73	1.10	-0.733	26.0	.0082**	-0.6788
rppgnet [9]	0.73	1.10	-0.366	65.0	.0385*	-0.3830
POS [5]	0.37	0.73	0.000	35.0	.0427*	-0.6106
GREEN [21]	0.73	1.10	-0.366	32.0	.0093**	-0.6333
ICA [23]	0.73	1.83	-0.732	87.5	.1649	-0.2124
CHROM [6]	0.37	1.10	-0.732	11.5	.0004***	-0.7888

$^{*}p < 0.05$
$^{**}p < 0.01$
$^{***}p < 0.001$

Supplementary Table 4 Wilcoxon Signed-Rank Test results for deep learning rPPG methods under different illumination scenarios on the CHILL dataset, grouped by training dataset. For each method, the median error under each condition is reported along with the median of the paired differences (Δ Median), test statistic (W), P-value, and effect size (r).

**Trained.**	**Model**	**Median Error**		**Δ Median**	**W-statistic**	**P-value**	**Effect Size (r)**
		**Bright**	**Dark**				
PURE [13]	DeepPhys [7]	0.73	0.73	0.000	304.0	.8545	-0.359
	Tscan [25]	0.73	0.73	0.000	381.5	.9048	-0.229
	Physnet [8]	13.18	11.73	1.098	348.0	.1309	-0.285
	rPPGNet [9]	9.52	15.02	-5.493	366.0	.0872	-0.254
COHFACE [17]	DeepPhys [7]	1.10	0.73	0.000	249.0	.3984	-0.452
	Tscan [25]	1.10	0.73	0.000	149.0	.2156	-0.620
	Physnet [8]	8.06	1.10	5.859	123.5	.00001***	-0.663
	rPPGNet [9]	5.86	20.14	-10.986	170.5	.00009***	-0.584

$^{***}p < 0.001$

Supplementary Table 5 Wilcoxon Signed-Rank Test results for deep learning rPPG methods under different HR conditions, grouped by datasets used for training. For each method, the median error under each condition is reported along with the median of the paired differences (Δ Median), test statistic (W), P-value, and effect size (r).

**Trained.**	**Model**	**Median Error**		**Δ Median**	**W-statistic**	**P-value**	**Effect Size (r)**
		**Low-HR**	**High-HR**				
PURE [13]	DeepPhys [7]	0.366	0.732	-0.366	21.0	.0040**	-0.717
	Tscan [25]	0.732	0.732	-0.366	47.0	.0246*	-0.520
	Physnet [8]	6.226	19.043	-13.55	0.0	.0001***	-0.876
	rPPGNet [9]	12.085	10.986	1.099	121.0	.5758	0.042
COHFACE [17]	DeepPhys [7]	1.465	0.366	-0.732	3.0	.0004***	-0.853
	Tscan [25]	1.099	0.732	-0.732	11.0	.0016**	-0.793
	Physnet [8]	5.493	1.831	-4.028	38.0	.0062*	-0.588
	rPPGNet [9]	10.254	16.113	10.254	179.0	.9864	0.482

*$p < 0.05$

**$p < 0.01$

***$p < 0.001$
